# Editorial: Discourse, conversation and argumentation: Theoretical perspectives and innovative empirical studies, volume II

**DOI:** 10.3389/fpsyg.2023.1141009

**Published:** 2023-01-24

**Authors:** Antonio Bova, Carlo Galimberti, Francesco Arcidiacono, Lise Haddouk

**Affiliations:** ^1^PSICOM–Centro Studi e Ricerche di Psicologia della Comunicazione, Catholic University of the Sacred Heart, Milan, Italy; ^2^Research Department, Haute Ecole Pédagogique des Cantons de Berne francophone, Biel/Bienne, Switzerland; ^3^Department of Psychology, Université de Rouen, Mont-Saint-Aignan, France

**Keywords:** argumentation, communicative interactions, conversation, discourse, psychology

Although discursive, conversational, and argumentative interactions play an essential role in our lives, there is no integrated area of psychological research on these types of communicative interactions (Arcidiacono et al., 2021). Research on discourse, conversation, and argumentation is conducted in several separate research communities that are spread across disciplines and have only limited intertwinement. This second volume of the Research Topic “*Discourse, Conversation and Argumentation: Theoretical Perspectives and Innovative Empirical Studies*” intends to offer a comprehensive dialogical platform to explore further novel and promising theoretical perspectives to study discursive, conversational, and argumentative interactions from a psychological perspective. In addition, it provides an extensive stand of the latest innovative research investigating discursive, conversational, and argumentative interactions between individuals, groups, and institutions to shed light on the most recent methodological developments in examining these types of interactions.

The Research Topic has thus permitted researchers working in different international psychological contexts to draw together their work within a common forum. Through their contributions, authors present an innovative state-of-the-art of collective evidence in psychological research on discourse, conversation, and argumentation. The numerous contributions focus on psychological perspectives on interactions and empirically supported approaches to analyze them. This panel of outstanding researchers contributes to rendering this second volume of this Research Topic particularly timely and open to colleagues continually exposed to nearly limitless sectorial approaches. In this vein, we hope that the contributions can be challenging for a large scientific audience to support integrated psychological research on discursive, conversational, and argumentative interactions.

The original research proposed by Liu and Zeng analyzes the key syntactic functions and basic grammatical meanings of utterance-middle pragmatic particle *dai* from syntactic positions, utterance functions, and prosodic features. This study demonstrates that *dai* is an overt marker of speakers' negative affective stance for complaining and criticizing in conversation. Meanwhile, due to the effects of high frequency and speakers' psychological cognition, also with the prosody, syntax, sequential positions, participants and interactional aims, there are also speakers' stance for sarcasm, surprise, and sympathy arising from *dai* though speakers' complaints and criticisms exist in varying degrees.

The paper of Brocos et al. presents original research offering a better understanding of how the epistemic and socio-relational dimensions of students' argumentative interactions are intertwined. By adopting discourse analysis to examine the interactions in a small group of four 11th-graders, the authors show that students, driven by epistemic aims in high socio-cognitive tension contexts, can refine the conditions they engage in argumentation.

Lu proposes a study on the use, function, and understanding of extended metaphors in L2 argumentative essays by Chinese learners of English. The function of extended metaphors is analyzed by adopting the bottom-up approach of establishing systematic metaphors from those identified extended metaphors to draw learners' communicative intentions in producing extended metaphors. This article contributes to the knowledge of learners' metaphoric competence in L2, which can, in turn, enrich teachers' metaphor knowledge and draw teachers' attention to learners' creative ways of using metaphors and then raise metaphor awareness in L2 writing, teaching, and learning.

Ghanbari and Salari propose a study on multiple data sources: students' perceptions of the argumentative texts, writing teachers' views on the students' argumentative writing, and analysis of the structure of the argumentative texts written by the students. Their investigation shows that the failure to develop an argumentative essay by the Iranian undergraduate English majors entails several academic, contextual, and pedagogical grounds.

The paper proposed by Qin and Wang considers the role of teachers' multimodal competence reflected through their multimodal pedagogic discourse in realizing the ultimate goals of classroom lead-ins. By exploring how two-winner teachers utilize their multimodal ensembles of communicative modes to engage students during classroom lead-ins, the authors show that different communicative modes construct the higher-level action of lead-in and are orchestrated into multimodal ensembles for the specific function of each lead-in move.

Another original research is proposed by Ji and Zhang, who analyze irregular self-selection in Chinese postgraduate EFL learners' conversations from the perspective of multimodal interaction. The authors use multimodal conversation analysis to investigate the detailed process of irregular self-selection. The paper contributes to understanding the detailed process of speakership claims in EFL learners' conversations.

The paper proposed by Munir Hashmi et al. attempts to provide insights into the argumentation structures in the discussion of Islam on social media involving 14 former Malaysian Muslims. The authors observe that the arguments put forth by former Muslims are loosely constructed rather than attempts to build a robust cumulative argumentation to support their reasons for abandoning the Muslim faith.

The interactive functions of questions in embodied collaborative work involving the manipulation of physical objects are investigated in the paper proposed by Bietti and Bietti. The authors conducted a systematic qualitative analysis of a dataset of 1,751 question-answer sequences to investigate how the questions identified are associated with accomplishing interactional goals and complementary temporalities in collaborative activities.

The opinion article by Rubinelli et al. points out that it is time for health institutions to invest in persuasive communication to combat low-quality information. Persuasive communication should show why institutional recommendations are worth being considered, as a way to provide important information that people can consider to engage in informed decision-making. In particular, according to the authors, it is fundamental that health institutions do not communicate to people “top-down,” but present their views using sound argumentation and showing precisely the ground of their claims.

The paper proposed by Shan explores the interaction between the prosodic and pragmatic characteristics of the discourse marker *ni zhidao* (“you know”) in spoken Chinese using instrumental methods. This study breaks through the limitations of traditional discourse marker research, which mainly relies on context and discourse characteristics for subjective reasoning, showing not only the part of *ni zhidao* in dynamically constructing and embodying specific contexts but also its communicative functions and the underlying meta-pragmatic awareness behind it.

Another original research is proposed by Wlodarczak and Heldner, who revisits the problem of breathing cues used to manage speaking turns in multiparty casual conversation. The authors propose a new categorization of turn-taking events that combines the criterion of speaker change with whether the original speaker inhales before producing the next talk spurt. This study highlights how the breathing signal can thus be successfully used for uncovering hidden turn-taking events, which are otherwise obscured by silence-based representations of interaction.

Esbo Agergaard and Nielsen conducted a discourse analysis of posts, comments, and contextual material on three Danish Facebook Pages, all established because of social groups' skepticism of human papillomavirus (HPV) vaccination. Based on a discourse analysis framework, this study shows that HPV vaccination skepticism is mediated through personal, epistemological, social, political, and value-laden discourses. Dealing with one of these dimensions alone, for example, treating HPV vaccination skepticism as an information deficit or a partisan issue, may risk missing the point entirely.

The paper of Aanesen et al. presents original research exploring how the female athletic body is constructed in the pseudonymous contemporary women's fitness magazine “Xrzise.” Based on a modified version of Parker's Foucauldian discourse analysis, this study shows that the female athletic body results from a complex nexus of different discourses associated with the powers of economy, sex differences, institutions, and ideological forces.

Heitmann et al. present a study on students' beliefs about the relevance of discourse and the role of facts. The authors highlight that students perceived the role of facts as highly relevant for science lessons, whereas discursive characteristics were considered significantly less important. This study lends further evidence to the existence of disciplinary school cultures in argumentation that may result from differences in teachers' school-track-specific classroom practice and education.

The paper proposed by Hansen considers whether young children understand that others may hold false beliefs, proposing a novel account of the logic of conversations about certain mental states. This study provides an innovative contribution to one of the most debated topics in psychology and neuroscience, supporting the view that even young children construe others in adult-like psychological terms.

## Author contributions

All authors listed have made a substantial, direct, and intellectual contribution to the work and approved it for publication.
